# The effect of chemical treatment of the sheep embryo zona pellucida on the ability of blastocysts to hatch after vitrification and warming

**DOI:** 10.1002/vms3.632

**Published:** 2021-09-16

**Authors:** Fatemeh‐Sadat Mousavi, Ebrahim Ahmadi, Abolfazl Shirazi, Naser Shams‐Esfandabadi, Hassan Nazari

**Affiliations:** ^1^ Department of Clinical Sciences Faculty of Veterinary Medicine Shahrekord University Shahrekord Iran; ^2^ Research Institute of Animal Embryo Technology Shahrekord University Shahrekord Iran; ^3^ Reproductive Biotechnology Research Center Avicenna Research Institute ACECR Tehran Iran

**Keywords:** acidic Tyrode's solution, blastocyst hatching, cryopreservation, pronase, zona pellucida

## Abstract

**Background:**

The embryo release from the zona pellucida is of prerequisites of successful implantation.

**Objectives:**

Regarding the negative impact of embryo cryopreservation on the blastocysts hatchability, the aim of the present study was to investigate the effects of treating embryonic zona pellucida with pronase or acidic Tyrode's solution (ATS) before morula formation on the viability, freezability, and hatchability of vitrified‐warmed resulted blastocysts.

**Methods:**

In the first experiment, the zona pellucida of 3‐ and 4‐day‐old embryos were treated with the above compounds for 30 or 45 s. Then, the competency of the treated embryos to reach to blastocyst stage and the hatchability of resulting blastocysts were investigated. In the second experiment, the cryo‐survivability and hatching rate of blastocysts resulting from 3‐day‐old embryos treated with pronase and ATS for 30 s were tested.

**Results:**

In the first experiment and in contrast to the 45 s exposure, 30‐s exposure of embryos to pronase or ATS did not have negative effect on the viability and development of embryos to blastocyst stage. In the second experiment, the freezability of blastocysts derived from 3‐day‐old embryos treated with pronase and ATS for 30 s was not different from that of the control group. However, the hatching rate of the pronase group was significantly higher than that of the control group.

**Conclusion:**

The results of the present study showed that reducing the thickness of zona pellucida of sheep embryos with pronase had no negative effect on the developmental competency and freezability of the treated embryos and improved the hatchability of vitrified‐warmed blastocysts.

## Abstract 2

In this study, we show that treating the zona pellucida of ovine embryos with pronase at cleavage stage improves the hatchability of resulting blastocysts after vitrification and warming. This is significant because may have practical application and improve the implantation rate of IVP vitrified‐warmed embryos.

## 1 INTRODUCTION

The release of the embryo from the zona pellucida is of prerequisites of successful implantation (Zhu et al., 1996). Under normal conditions, the oocyte is covered with a highly specialized extracellular glycoprotein coat and its structure changes after the first sperm enters the oocyte to prevent polyspermy (Bansal & Gupta, 2009; Martins et al., 2011). In addition to protecting the oocyte and embryo, zona pellucida prevents the separation of blastomeres from each other and also probably plays a role in guiding the embryo to the uterus (Bansal & Gupta, 2009; Sifer et al., 2006). As the embryo continues to grow and reach the blastocyst stage and expands, the zona pellucida becomes thinner and prepares for a cleft (Ebner et al., 2005; Hagemann et al., 2010; Sifer et al., 2006). In sheep on the sixth and seventh days after fertilization, the hatching process occurs. Blastocyst hatching allows the embryo to attach the endometrium. Any changes that disrupt the hatching process will seriously affect fertility (Aktan et al., 2006; Sifer et al., 2006).

Relatively low level of blastocyst hatching is a common problem in in vitro embryos, one of the reasons for which is zona hardening. In cases where fertilization and culture of the embryo occur in in vitro conditions, some environmental stresses such as temperature and pH of the culture medium (Santos Filho et al., 2010) can increase its elasticity by changing the structure of the zona, which prevents embryo from hatching and implantation (Hiraoka et al., 2009; Hur et al., 2011). Another cause of zona hardening in laboratory embryos is the cryopreservation process. The process of embryo cryopreservation and the subsequent prolongation of embryo culture increase the hardness of zona (Zech et al., 2005). On the other hand, the energy shortage of the cryopreserved embryos which results in the reduction of Na^+^/K^+^‐ATPase pump activity is mentioned as one of the factors delaying the return of blastocyst to its original state and also disrupting its hatching because the activity of this pump is responsible for the blastocyst expansion and consumes more than 60% of embryonic energy in the blastocyst stage (Iwayama et al., 2011; Nagy et al., 2005).

According to the abovementioned introduction, it seems that reducing the thickness of zona pellucid could be helpful in the process of blastocyst hatching (Sifer et al., 2006). Various methods have been used to pierce and reduce the thickness of the zona, including the use of acidic Tyrode's solution (ATS), enzymatic digestion, and the use of laser systems (Balaban et al., 2006; Ebner et al., 2005; Edwards, 2007; Petersen et al., 2005). In general, regional thinning or perforating an area of zona pellucida in human embryos improves the rate of embryo hatching and implantation (Nagy et al., 2005).

To the best of our knowledge, the effect of embryonic zona pellucida thinning on the hatchability of farm animals cryopreserved blastocysts has not been investigated well. Therefore, the aim of the present study was to investigate the effects of treating the zona pellucida of ovine embryos with pronase enzyme or ATS before morula formation on the viability and developmental competence of treated embryos and on the freezability and post‐warming hatchability of resulted blastocysts.

## 2 MATERIALS AND METHODS

All chemicals were obtained from Sigma Chemicals Co. (St. Louis, MO, USA) unless otherwise stated.

### 2.1 In vitro embryo production

The ovaries of slaughtered ewes were transported to the laboratory in normal saline solution containing antibiotics (200 IU/ml penicillin and 0.2 mg/ml streptomycin) at 25–35°C. In the laboratory, the ovaries were washed with tap water and then placed in saline containing antibiotics in a water bath at 38°C. Follicular fluids of all visible 2–6 mm follicles were aspirated into a 50 ml conical tube containing HEPES‐buffered M199 plus 10% FBS and 100 IU/ml heparin. Under a stereomicroscope, cumulus‐oocyte complexes (COCs) were separated from the aspirate using pulled pasteur pipettes and rinsed three times in HEPES‐buffered M199 plus 10% FBS. Ten good quality COCs (oocytes with even granular cytoplasm and at least three layers of dense cumulus cells around their zona pellucid) were cultured in mineral oil‐covered 50 μl droplets of maturation medium and incubated in an incubator at 39°C and 5% CO_2_ in humid atmosphere for 22–24 h. The maturation medium was bicarbonate‐buffered M199 containing 10% FBS and 0.05 IU/ml FSH.

After in vitro maturation, the oocytes were transferred to the droplets of IVF‐TALP and incubated at 39°C with 5% CO_2_ in humidified atmosphere until sperm addition. The spermatozoa were prepared according to Hajihassani et al. (2019). Briefly, 200 μl of frozen‐thawed epididymal sperm suspension was placed on 1 ml Histoprep and centrifuged at 300 × *g* for 5 min. The pellet of spermatozoa after re‐suspension in the 50 μl of Sperm‐TALP was added to the fertilization droplets at the final concentration of 1 × 10^6^ motile sperm cells/ml. At 24–26 h post‐insemination, five to six presumptive zygotes were cultured in the 20 μl droplets of IVC‐SOF medium (SOF+amino acids and BSA) in an incubator with 5% CO_2_ and 6% O_2_ at 39°C with maximum humidity. Cleaved embryos were separated 48 h after IVC and cultured in IVC‐SOF medium supplemented with 10% charcoal striped FBS. The rate of blastocyst formation and hatching was recorded on day 7 after culture (Day 0 = IVF).

### 2.2 Vitrification and warming

The vitrification and warming of blastocysts were performed according to Shirazi et al. (2010) with some modifications. Briefly, the base medium for preparation of all vitrification and warming solutions was HEPES‐buffered M199 supplemented with 20% (v/v) FBS. Vitrification was performed at room temperature (approximately 25°C). Three expanding 6‐day‐old blastocysts were placed in a 100 μl droplet of base medium (20–30 s) and were then transferred to the equilibration medium (7.5% ethylene glycol + 7.5% DMSO) for 5 min. Then, blastocysts were transferred to a 100 μl droplet of vitrification solution (15% ethylene glycol + 15% DMSO + 0.5 M sucrose). The embryos were then loaded with a fine bore pasture pipette on a cryotop (Kitazato Ltd. Tokyo, Japan) and directly plunged into the liquid nitrogen. The total incubation time of blastocysts in the vitrification solution including loading on the cryotops and plunging in the liquid nitrogen was 30 seconds. For warming, the tip of the cryotops was directly immersed into a 100 μl droplet of warming solution (base medium + 0.5 M sucrose) at 37°C. After 5 min, blastocysts were washed two times in the base medium. The vitrified/warmed blastocysts were then cultured in IVC‐SOF medium for 72 h. The survival and hatching of blastocyst were recorded at 24 and 72 h post‐warming, respectively.

### 2.3 Experimental design

This study had two experiments. The first experiment was performed to assess the developmental competence of embryos treated with pronase and ATS (Sigma T1788, pH 2.5) and also, to evaluate the effect of the time of exposure of embryos to these compounds. In this experiment, on days 3 and 4 after the start of embryo culture (IVF = day 0), embryos that had a normal morphology and was not arrested were isolated and randomly divided into experimental groups: control, pronase 30 and 45 s, and ATS 30 and 45 s.

To treat with ATS, a 75 μl droplet of ATS was placed in the centre of a 6 cm petri dish, and 10 droplets of 75 μl HSOF containing 4 mg BSA were placed around it as washing drops and then covered with oil. The embryos in groups of 2–3 were first placed in the centre droplet for 30 or 45 seconds and then transferred to the first washing droplet. The embryos were placed in the first three drops of washing medium for 10 s each and in the next droplets for 30 s each. During the transfer of embryos between the droplets, the least amount of medium was transferred with them. Finally, the embryos were collected in the last droplet. To treat with pronase, by adding HTCM culture medium containing 10% serum to a pre‐prepared aliquot, a 0.25% (w/v) working solution of the enzyme was prepared (Nazari et al., 2016). Then, in the centre of a petri dish, several droplets of 10 μl of the enzyme and around them 20 droplets of 20 μl of HTCM medium containing 20% serum were placed as washing droplets and covered with oil. Two to three embryos were transferred to one of the enzyme droplets for 30 or 45 s. Then, the embryos were transferred to a rinsing droplet. In the first few droplets, the embryos were immediately transferred to the next droplet to stop the enzyme activity. During the transfer of embryos between the droplets, the least amount of medium was transferred with them. In total, each group of embryos was washed in 10 washing droplets and all were collected in one droplet. Finally, after treating all embryos, they were transferred to IVC droplets and cultured up to day 7 (Day 0 = IVF) on which the rate of blastocyst formation and hatching was recorded.

The second experiment was performed to evaluate the freezability and post‐warming hatchability of the blastocysts derived from embryos treated with the above compounds. Based on the results of the first experiment, 3‐day‐old embryos were exposed to pronase and ATS for 30 s and then cultured as in the first experiment. On day 6 of IVC, expanding blastocysts of pronase, ATS, and control (without treatment) groups were vitrified and warmed.

### 2.4 Statistical analysis

The data were analyzed by Chi‐square test or Fisher's exact test, when appropriate. Statistical analysis was performed using IBM‐SPSS version 22 software package. Differences were considered significant at the level of *p* < 0.05.

## 3 RESULTS AND DISCUSSION

The present study aimed to overcome low hatchability of cryopreserved IVP blastocysts. We treated ovine cleavage stage embryos with two zona digesting agents and evaluated their post‐treatment development and the cryo‐survival and post‐thawing hatchability of the resulted blastocysts. We hypothesized weakening the zona pellucida structure could ease the expansion and hatching of vitrified‐warmed blastocysts.

At first, cleavage stage embryos on the third and fourth days of culture were treated with pronase and ATS to elucidate the possible toxic effect of the agents on the embryonic development and to find the optimum exposure time. The results are presented in Table 1. Since the removal of embryonic zona pellucida before morula compaction leads to the dispersion and loss of blastomeres, we did not want complete zona disintegration. Although a recognizable zona thinning was observed at 45 s exposure of 3‐day‐old embryos to the zona digesting agents, this exposure time was clearly harmful to the treated embryos. In the ATS treated embryos, 45 s‐exposure led to a decreased viability (degeneration) and reduced blastocyst development. In pronase treated embryos, 45 s of exposure also resulted in the degeneration of some embryos. Moreover, as the zona digesting activity of the enzyme did not terminate immediately after transferring embryos from the enzyme droplet to washing droplets, exposure for 45 s led to the zona pellucida disintegration and blastomeres dispersion in some embryos and hence, overall embryonic viability decreased. Due to the deleterious effect of 45 s‐exposure on 3‐day‐old embryos, this time was not tested on 4‐day‐old embryos.

**TABLE 1:** The effect of treating day 3 or 4 embryos with acidic Tyrode's solution (ATS) and pronase for 30 and 45 s on embryonic development

Treatments	Exposure time (s)	Embryonic age (days)	Number of treated embryos	Survived embryos^*^ (%)	Blastocysts (% of treated embryos)	Hatched blastocysts (%)
ATS	30	3	25	21 (84)^a^	10 (40)^abc^	5 (50)^ab^
	30	4	24	21 (87.5)^a^	10 (41.7)^ac^	5 (50)^ab^
	45	3^#^	25	12 (48)^b^	3 (12)^b^	0 (0)^a^
Pronase	30	3	27	25 (92.6)^a^	13 (48.1)^c^	9 (69.2)^ab^
	30	4	25	23 (92)^a^	12 (48)^c^	6 (50)^ab^
	45	3	25	13 (52)^b^	5 (20)^ab^	5 (100)^b^
Control	–	3	35	35 (100)^a^	19 (54.3)^c^	10 (52.6)^ab^

*Note*: Different letters in each column indicate a significant difference (*p* < 0.05).

^*^The post‐treatment survival of embryos was evaluated after 24 h culture.

^#^Due to the destructive effects of 45 s exposure to both compounds, this time was not tested on 4‐day‐old embryos.

As shown in Table 1, the viability, blastocyst rate, and blastocyst hatching rate of the embryos that were exposed to the zona digesting agents for 30 s were not significantly different from those of the control (untreated) group. Moreover, blastomeres dispersion was not observed in 30 s‐pronase‐treated embryos. Since the results of the treating embryos on the third and fourth days were not different, only the 3‐day‐old embryos were treated for performing the second experiment. The hatching rate of the pronase‐treated 3‐day‐old embryos was slightly better than those of the other groups and 100% of blastocysts of 45 s‐pronase‐treated group hatched, but due to the overall decrease of blastocyst rate, this time was not tested in the second experiment.

Assisted hatching has been considered as a way to overcome poor pregnancy rates following embryo transfer in bovine (Taniyama et al., 2011) and humans (Hammadeh et al., 2011). Various methods have been used for assisted hatching, including mechanical, chemical, enzymatic methods, and laser beams (Balaban et al., 2006; Petersen et al., 2005). In bovine, embryonic zona pellucida cutting using a needle under either an inverted microscope or a stereomicroscope resulted in improved pregnancy rates following transferring poor quality in vivo produced embryos (Taniyama et al., 2011). In the current study, pronase and ATS were chosen because they are cheap and easy to apply and can be used in laboratories equipped with the least types of equipment required for an embryology laboratory. Cleavage stage embryos were treated to give the embryos ample opportunity to repair any damage caused by exposure to the enzyme or ATS solution. Similarly, vitrified‐warmed blastocysts were not treated to avoid exerting additional stress which could compromise their post‐warming recovery. As mentioned earlier, our aim was also to select a time of exposure to zona digestive compounds in such a way that while removing the greatest amount of zona thickness, it does not damage its integrity and has the least adverse effect on fetal development.

Our results showed that treating ovine 3‐day‐old embryos with pronase for 30 s had the least negative effect on the developmental capacity of treated embryos. Although the hatching rate of fresh embryos was similar to untreated embryos, vitrified‐warmed blastocysts derived from pronase‐treated embryos had a significantly higher hatching rate than that of the untreated control group (*p* = 0.049, Table 2). Moreover, our results are in accordance with Taniyama et al. (2014) in which the hatchability of bovine fresh and frozen‐thawed in vitro derived blastocyst was improved by treating them with pronase at morula stage. ATS did not affect the post‐warming blastocyst hatchability. Treating embryos with pronase is simple and fast and can be applied by technicians who have minimal embryo handling skills. Partial chemical zona thinning by ATS applied by micromanipulator was superior to circumferential zona thinning (Yano et al., 2007), since it needs a micromanipulator, it cannot be applied in every laboratory. Therefore, circumferential zona thinning by pronase according to our protocol may be a good option for improving the pregnancy rate of transferred embryos in farm animals.

**TABLE 2:** Results of vitrification and warming of blastocysts derived from 3‐day‐old embryos treated with acidic Tyrode's solution (ATS) and pronase for 30 s

Treatment	Number of treated embryos	Survival after treatment (%)	Blastocyst (% of treated embryos)	Number of vitrified blastocysts	Survival after warming (%)	Hatched blastocyst (%)
ATS	70	65 (92.9)	30 (42.9)	25	16 (69.6)	7 (43.8)^ab^
Pronase	73	69 (94.5)	34 (46.6)	28	19 (67.9)	14 (73.7)^a^
Control	55	55 (100)	29 (52.7)	25	18 (72)	7 (38.9)^b^

*Note*: Different letters in each column indicate a significant difference (*p* < 0.05).

## CONFLICT OF INTEREST

The authors declare no conflict of interest.

## ETHICS STATEMENT

The authors confirm that the ethical policies of the journal, as noted on the journal's author guidelines page, have been adhered to. No ethical approval was required as abattoir derived materials were used in the study.

We declare no one of the authors listed on the manuscript are employed by an Iran's government agency that has a primary function other than research and/or education. Moreover, we also declare no one of the authors are submitting this manuscript as an official representative or on behalf of the Iran government.

## AUTHOR CONTRIBUTIONS

*Investigation (lead) and writing‐original draft (supporting)*: Fatemeh Sadat Mousavi. *Conceptualization (lead), formal analysis, project administration, supervision (lead), writing‐original draft (lead), and writing‐review & editing (lead)*: Ebrahim Ahmadi. *Conceptualization (supporting), supervision (supporting), and writing‐review & editing (supporting)*: Abolfazl Shirazi. *Resources and writing‐review & editing (supporting)*: Naser Shams‐Esfandabadi. *Investigation (supporting) and writing‐review & editing (supporting)*: Hassan Nazari.

### PEER REVIEW

The peer review history for this article is available at https://publons.com/publon/10.1002/vms3.632

## Data Availability

The author has provided the required data availability statement and, if applicable, included functional and accurate links to said data therein.

## References

[vms3632-bib-0001] Aktan, E. , Ozer, D. , Demirol, A. , Gurgan, T. , & Bozkurt, K. (2006). The effect of zona thinning size on implantation and pregnancy rates of ICSI‐ET patients with advanced woman age. Middle East Fertility Society Journal, 11(3), 183–190.

[vms3632-bib-0002] Balaban, B. , Urman, B. , Yakin, K. , & Isiklar, A. (2006). Laser‐assisted hatching increases pregnancy and implantation rates in cryopreserved embryos that were allowed to cleave in vitro after thawing: a prospective randomized study. Human Reproduction, 21(8), 2136–2140. 10.1093/humrep/del097 16613888

[vms3632-bib-0003] Bansal, P. , & Gupta, S. (2009). Binding characteristics of sperm with recombinant human zona pellucida glycoprotein‐3 coated beads. Indian Journal of Medical Research, 130(1), 37–43. 19700799

[vms3632-bib-0004] Ebner, T. , Moser, M. , & Tews, G. (2005). Possible applications of a non‐contact 1.48 μm wavelength diode laser in assisted reproduction technologies. Human Reproduction Update, 11(4), 425–435. 10.1093/humupd/dmi009 15817523

[vms3632-bib-0005] Edwards, R. G. (2007). IVF, IVM, natural cycle IVF, minimal stimulation IVF–time for a rethink. Reproductive BioMedicine Online, 15(1), 106–119. 10.1016/S1472-6483(10)60699-2 17623547

[vms3632-bib-0006] Hagemann, A. R. , Lanzendorf, S. E. , Jungheim, E. S. , Chang, A. S. , Ratts, V. S. , & Odem, R. R. (2010). A prospective, randomized, double‐blinded study of assisted hatching in women younger than 38 years undergoing in vitro fertilization. Fertility and Sterility, 93(2), 586–591. 10.1016/j.fertnstert.2009.01.116 19268926

[vms3632-bib-0007] Hajihassani, A. , Ahmadi, E. , Shirazi, A. , & Shams‐Esfandabadi, N. (2019). Reduced glutathione in the freezing extender improves the in vitro fertility of ram epididymal spermatozoa. Small Ruminant Research, 174, 13–18. 10.1016/j.smallrumres.2019.02.016

[vms3632-bib-0008] Hammadeh, M. E. , Fischer‐Hammadeh, C. , & Ali, K. R. (2011). Assisted hatching in assisted reproduction: A state of the art. Journal of Assisted Reproduction and Genetics, 28(2), 119–128. 10.1007/s10815-010-9495-3 21042844 PMC3059528

[vms3632-bib-0009] Hiraoka, K. , Hiraoka, K. , Horiuchi, T. , Kusuda, T. , Okano, S. , Kinutani, M. , & Kinutani, K. (2009). Impact of the size of zona pellucida thinning area on vitrified‐warmed cleavage‐stage embryo transfers: A prospective, randomized study. Journal of Assisted Reproduction and Genetics, 26(9–10), 515–521. 10.1007/s10815-009-9350-6 19830543 PMC2788686

[vms3632-bib-0010] Hur, Y. S. , Park, J. H. , Ryu, E. K. , Yoon, H. J. , Yoon, S. H. , Hur, C. Y. , Lee, W. D. , & Lim, J. H. (2011). Effect of artificial shrinkage on clinical outcome in fresh blastocyst transfer cycles. Clinical and Experimental Reproductive Medicine, 38(2), 87–92. 10.5653/cerm.2011.38.2.87 22384424 PMC3283060

[vms3632-bib-0011] Iwayama, H. , Hochi, S. , & Yamashita, M. (2011). In vitro and in vivo viability of human blastocysts collapsed by laser pulse or osmotic shock prior to vitrification. Journal of Assisted Reproduction and Genetics, 28(4), 355–361. 10.1007/s10815-010-9522-4 21152966 PMC3114962

[vms3632-bib-0012] Martins, W. P. , Rocha, I. A. , Ferriani, R. A. , & Nastri, C. O. (2011). Assisted hatching of human embryos: A systematic review and meta‐analysis of randomized controlled trials. Human Reproduction Update, 17(4), 438–453. 10.1093/humupd/dmr012 21474527

[vms3632-bib-0013] Nagy, Z. P. , Taylor, T. , Elliott, T. , Massey, J. B. , Kort, H. I. , & Shapiro, D. B. (2005). Removal of lysed blastomeres from frozen–thawed embryos improves implantation and pregnancy rates in frozen embryo transfer cycles. Fertility and Sterility, 84(6), 1606–1612. 10.1016/j.fertnstert.2005.06.027 16359953

[vms3632-bib-0014] Nazari, H. , Shirazi, A. , Shams‐Esfandabadi, N. , Afzali, A. , & Ahmadi, E. (2016). The effect of amniotic membrane stem cells as donor nucleus on gene expression in reconstructed bovine oocytes. The International Journal of Developmental Biology, 60(4–6), 95–102. 10.1387/ijdb.160010hn 27389982

[vms3632-bib-0015] Petersen, C. G. , Mauri, A. , Baruffi, R. , Oliveira, J. , Massaro, F. C. , Elder, K. , & Franco Jr, J. (2005). Implantation failures: Success of assisted hatching with quarter‐laser zona thinning. Reproductive BioMedicine Online, 10(2), 224–229. 10.1016/S1472-6483(10)60944-3 15823228

[vms3632-bib-0016] Santos Filho, E. , Noble, J. , & Wells, D. (2010). A review on automatic analysis of human embryo microscope images. The Open Biomedical Engineering Journal, 4, 170–177. 10.2174/1874120701004010170 21379391 PMC3044885

[vms3632-bib-0017] Shirazi, A. , Soleimani, M. , Karimi, M. , Nazari, H. , Ahmadi, E. , & Heidari, B. (2010). Vitrification of in vitro produced ovine embryos at various developmental stages using two methods. Cryobiology, 60(2), 204–210. 10.1016/j.cryobiol.2009.11.002 19919830

[vms3632-bib-0018] Sifer, C. , Sellami, A. , Poncelet, C. , Kulski, P. , Martin‐Pont, B. , Bottero, J. , Porcher, R. , Cedrin‐Durnerin, I. , Hugues, J. , & Wolf, J. (2006). A prospective randomized study to assess the benefit of partial zona pellucida digestion before frozen‐thawed embryo transfers. Human Reproduction, 21(9), 2384–2389. 10.1093/humrep/del149 16772285

[vms3632-bib-0019] Taniyama, A. , Nishino, Y. , Inoue, T. , Taura, Y. , Takagi, M. , Otoi, T. , Sato, M. , & Kubota, C. (2014). Effects of chemical zona pellucida thinning with pronase on the development of bovine embryos. Journal of the Japan Veterinary Medical Association, 67(11), 833–838. 10.12935/jvma.67.833

[vms3632-bib-0020] Taniyama, A. , Watanabe, Y. , Nishino, Y. , Inoue, T. , Taura, Y. , Takagi, M. , Kubota, C. , & Otoi, T. (2011). Assisted hatching of poor‐quality bovine embryos increases pregnancy success rate after embryo transfer. Journal of Reproduction and Development, 57(4), 543–546. 10.1262/jrd.10-096T 21606632

[vms3632-bib-0021] Yano, K. , Yano, C. , Kubo, T. , Ohashi, I. , Maeda, N. , & Fukaya, T. (2007). Chemical zona pellucida thinning with acidified Tyrode's solution: Comparison between partial and circumferential techniques. Journal of Assisted Reproduction and Genetics, 24(10), 471–475. 10.1007/s10815-007-9131-z 17701000 PMC3455074

[vms3632-bib-0022] Zech, N. , Lejeune, B. , Zech, H. , & Vanderzwalmen, P. (2005). Vitrification of hatching and hatched human blastocysts: Effect of an opening in the zona pellucida before vitrification. Reproductive BioMedicine Online, 11(3), 355–361. 10.1016/S1472-6483(10)60844-9 16176678

[vms3632-bib-0023] Zhu, S. , Sakurai, T. , Edashige, K. , Machida, T. , & Kasai, M. (1996). Cryopreservation of zona‐hatched mouse blastocysts. Reproduction, 107(1), 37–42. 10.1530/jrf.0.1070037 8699432

